# Honokiol affects the composition of gut microbiota and the metabolism of lipid and bile acid in methionine-choline deficiency diet-induced NASH mice

**DOI:** 10.1038/s41598-023-42358-w

**Published:** 2023-09-14

**Authors:** Ting Zhai, Junjun Wang, Yong Chen

**Affiliations:** Hubei Province Key Laboratory of Biotechnology of Chinese Traditional Medicine, National and Local Joint Engineering Research Center of High-Throughput Drug Screening Technology, State Key Laboratory of Biocatalysis and Enzyme Engineering, Hubei University, Wuhan, 430062 China

**Keywords:** Diseases, Molecular medicine

## Abstract

Honokiol (HNK), one of the main active components of *Magnolia officinalis*, has a positive effect on non-alcoholic steatohepatitis (NASH). However, the effects of HNK on the composition of serum lipids and bile acids (BAs) and gut microbiota (GM) of NASH mice are still unknown.C57BL/6 mice were fed with methionine-choline deficiency (MCD) diet and gavaged with HNK (20 mg/kg/d) for 8 weeks, then the serum lipids and BAs were detected by LC–MS, the composition of ileum microflora and the mRNA expression of hepatic BAs homeostasis related genes were analyzed by 16S rDNA sequencing and RT-qPCR, respectively. HNK treatment decreased the degree of hepatic lipid drops, inflammatory cell infiltration and fibrosis. Meantime, the serum levels of 34 lipids and 4 BAs in MCD mice were significantly altered by HNK treatment, as well as the increased abundance of *Ruminococcaceae*, *Caulobacteraceae* and *Brevundimonas*, and the decreased abundance of *Firmicutes* and *Dubosiella*. Besides, HNK treatment increased the hepatic mRNA expression of *Oatp1b2* in MCD mice. The ameliorating effect of HNK on NASH may be partly related to its correction on the disorders of GM, serum lipids and BAs of MCD mice.

## Introduction

Non-alcoholic steatohepatitis (NASH) is an important manifestation of NAFLD progression characterized by steatosis, inflammation, and further fibrosis, culminating in cirrhosis and liver cancer. The pathogenesis of NASH remains uncertain. Liver fat deposition is the basic manifestation of NASH, and increased liver intake of free fatty acids (FFAs) and de novo synthesis of fatty acids, decreased lipid oxidative decomposition and relatively insufficient lipoprotein output can all lead to liver lipid deposition and promote NASH^1^. Lipid metabolism disorder causes excessive FFAs to reside in muscle, liver and adipose tissue, leading to IR, which in turn aggravates lipid metabolism disorder, forming a vicious cycle^2^. At the same time, lipid metabolism disorder also promotes the production of a large number of cytokines, such as tumor necrosis factor (TNF-α), interleukin (IL-6) and adiponectin, which induces oxidative stress and inflammatory response^3^. All of these contribute to the NASH process.

Recently, more and more evidences indicate that bile acids (BAs) and gut microbiota (GM) also act crucial roles in the occurrence, development and regression of NASH. BAs are a class of cholesterol derivatives synthesized mainly in the liver and enteric canal, and participate in the process of NASH through the FXR-SHP, FGF15(mouse)/19(human)-FGFR4 and TGR5 pathways^4^. Dysfunction of the enteric-liver axis, including changes in mucosal permeability, bacterial over-propagation and GM disorder, can significantly promote the occurrence and development of NAFLD^5^. Patients with NASH show slow intestinal peristalsis, destroyed intestinal villi and disordered GM^6^. Colonizing germ-free mice with cecal microbiota obtained from conventionally raised mice led to a 60% increase in body fat content and increased insulin resistance (IR) within 14 days despite reduced food intake^7^. Chronic oral administration of antibiotics effectively suppressed the gut bacteria, decreased portal secondary BAs levels, and attenuated hepatic inflammation and fibrosis in NAFLD mice^8^. These reports suggested that BAs and GM play an important role in the development of NASH.

Honokiol (HNK) is one of the main bioactive components of *Magnolia officinalis* which has antibacterial, anti-inflammatory, antiviral and anti-tumor effects^9^. Studies have shown that HNK can reduce the weight of white adipose tissue (WAT) and the size of adipose cells in obese mice fed by HFD, suppress the expression of WAT pro-inflammatory genes, and improve IR^10^. In FFAs-treated HepG2 cells and mice fed by HFD, HNK induced hepatic ACC phosphorylation and inhibited the maturation of sterol regulatory-element-binding protein-1c (SREBP-1c) by regulating LKB1-AMPK pathway, and ultimately alleviated lipid accumulation of liver cells^11^. Our previous studies also indicated that HNK not only inhibited the expression of SREBP-1C, PNPLA3, CYP2E1 and CYP4A, improved lipid accumulation and lipid peroxidation in liver cells with steatosis^12^, but also alleviated hepatic steatosis and oxidative stress of mice fed by methionine-choline deficiency (MCD) diet through regulating CFLAR-JNK pathway^13^.

In view of the important role of the enteric-liver axis in BAs and lipid metabolism and its close relationship with NASH, the present work studied the effects of HNK on the composition of lipids and BAs in serum, and GM in ileum of MCD mice by using metabolomics and 16S rDNA sequencing techniques, which provided useful reference for elucidating the mechanism of HNK against NASH.

## Results

### Effect of HNK on the hepatic biomarkers in the tested mice

**Figure 1:**
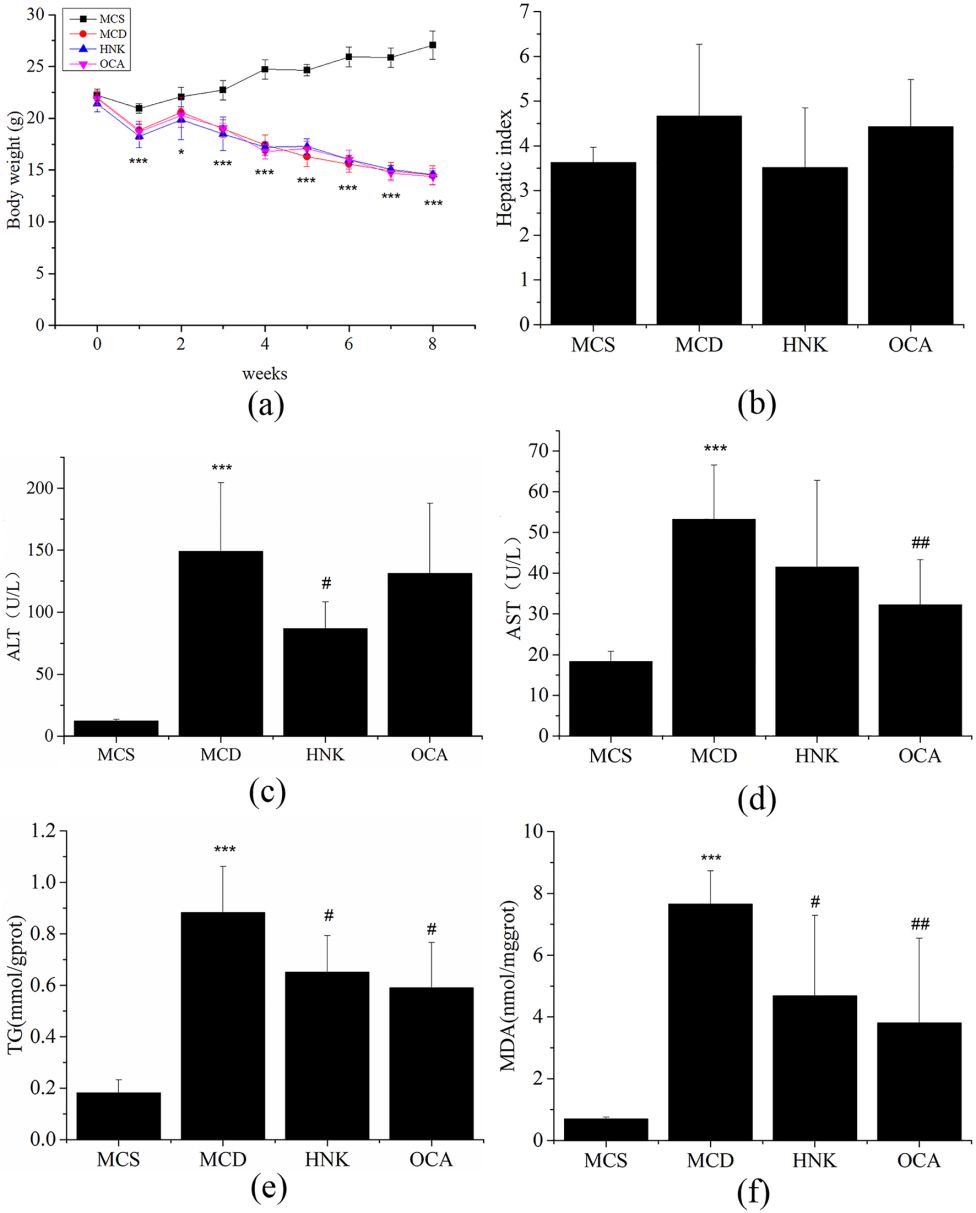
Effect of HNK on the hepatic biomarkers in the tested mice. Mice were fed with a methionine-choline sufficient (MCS) diet, a methionine-choline deficient diet (MCD), MCD plus HNK (20 mg/kg/d) or OCA (6.5 mg/kg/d) for 8 weeks. Changes in the (**a**) Body weight and (**b**) Hepatic index. Serum levels of (**c**) ALT, (d) AST; Liver levels of (**e**) TG, (f) MDA. Data were expressed as mean ± SD (n = 7). ***p < 0.001 versus MCS group; #*p* < 0.05, ##*p* < 0.01 versus MCD group. MCD, methionine- and choline-deficient diet; MCS, methionine- and choline-sufficient diet; HNK, honokiol; OCA, obeticholic acid.

Hepatic biomarkers of the mice were detected by commercial test kits. The results showed that HNK had no effect on weight loss and hepatic index of mice fed by MCD diet ((Fig. 1a and b). Compared with MCS group, serum ALT, AST and liver TG, MDA levels in MCD group were significantly increased. After HNK treatment, serum ALT activity, liver TG and MDA content decreased remarkably, AST level was reduced similarly, but there was no significant difference compared with MCD group. In addition, the levels of liver TG, MDA and serum AST in OCA group were obviously lower than those in MCD group (Fig. 1c–f).

### HNK attenuated hepatic steatosis, inflammation and fibrosis of NASH mice

**Figure 2:**
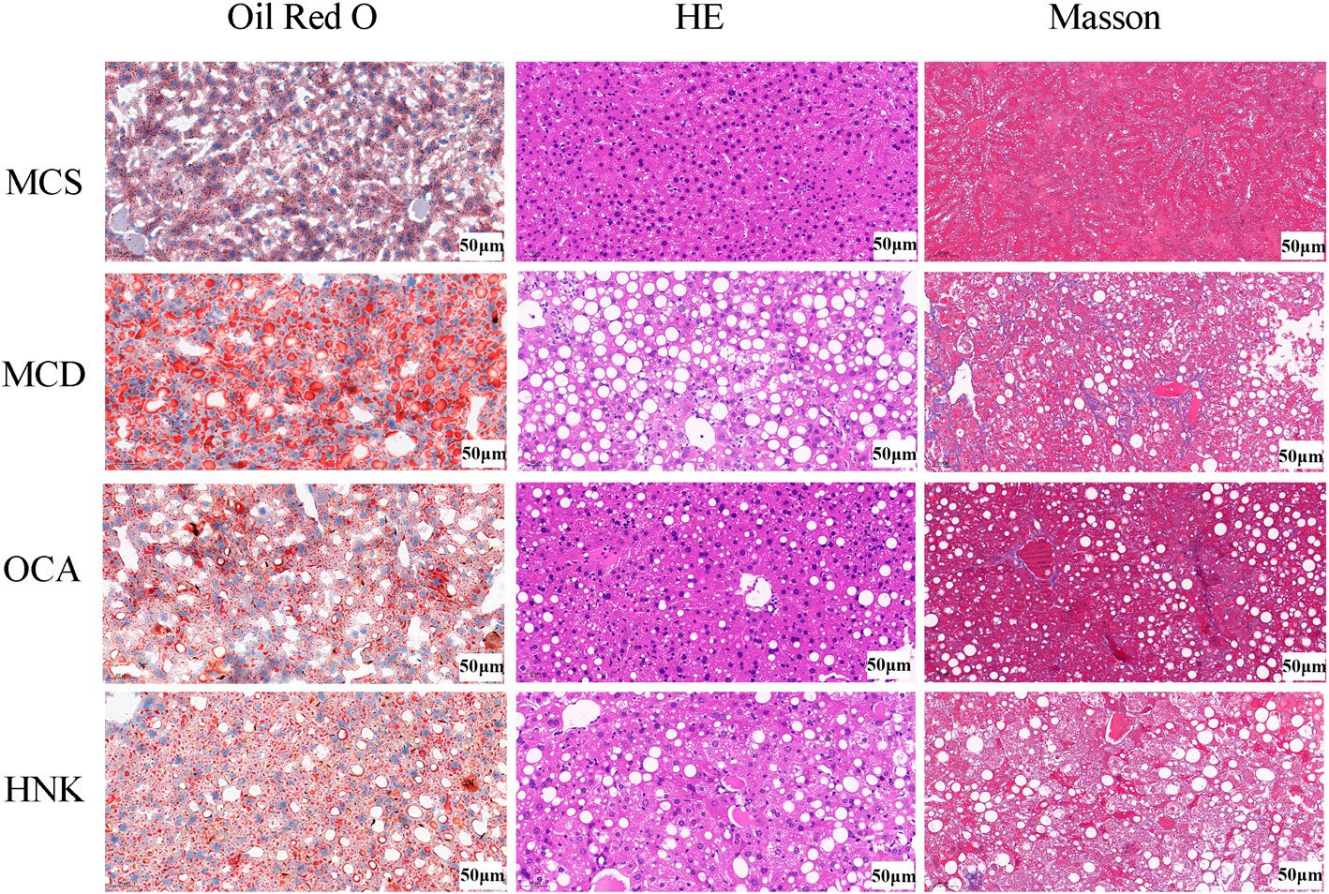
The hepatic histological changes of the tested mice evaluated by Oil Red O, HE and Masson staining (200 ×). HE, hematoxylin and eosin; MCD, methionine- and choline-deficient diet; MCS, methionine- and choline-sufficient diet; HNK, honokiol; OCA, obeticholic acid.

Hepatic lipid accumulation, steatosis, inflammation and fibrosis of the mice were analyzed by Oil red O staining, HE staining and Masson staining (Fig. 2). Compared with MCS group, the liver cells of MCD mice were disordered and showed ballooning changes, with a large number of lipid droplets and fat vacuoles, accompanied by inflammatory cell infiltration and fibrosis. After treatment with HNK and OCA, the number of lipid droplets and fat vacuoles in liver cells were reduced significantly, the degree of inflammatory cell infiltration and fibrosis decreased, and the liver tissue morphology was closer to MCS group.

### Effects of HNK on serum lipids of NASH mice

**Figure 3:**
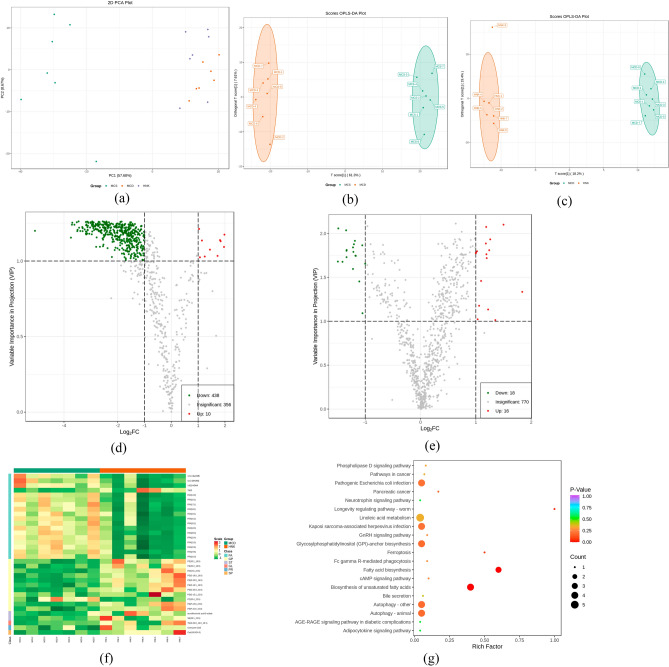
Model analysis of serum lipid metabolic profile in the tested mice (n = 7). (**a**) PCA score plot. (**b**) OPLS-DA score plot between MCS and MCD groups (*R*^2^*X*(cum) = 0.706,*Q*^2^(cum) = 0.967). (**c**) OPLS-DA score plot between MCD and HNK groups (*R*^2^*X*(cum) = 0.458,*Q*^2^ (cum) = 0.502). (**d**) Volcano plots of lipid metabolites between MCS and MCD groups. (**e**)Volcano plots of lipid metabolites between MCD and HNK groups. (**f**) Analysis of lipid metabolism pathway based on the significant differences of MCD verus HNK groups. (**g**) Lipid metabolic pathway analysis of differential lipid species between HNK and MCD groups (www.kegg.jp/kegg/kegg1.html). MCD, methionine- and choline-deficient diet; MCS, methionine- and choline-sufficient diet; HNK, honokiol.

The principal component analysis (PCA) showed the separation trend of serum lipid composition in each group. As shown in Fig. 3a, there was an obvious separation trend between the MCS group and the MCD group, indicating that MCD diet caused significant changes of serum lipid composition in mice. Compared with MCD group, the sample distribution of HNK group showed no change.

To further clarify the differential lipids among each group, we analyzed the serum lipid composition of MCS, MCD and HNK groups using orthogonal partial least squares discriminant analysis (OPLS-DA). As shown in Fig. 3b, MCS and MCD group were significantly separated. *R*^2^Y = 0.997, *Q*^2^ = 0.967, and both *R*^2^*Y* and *Q*^2^ were greater than 0.4, indicating that the OPLS-DA model was effective. Figure 3c showed that HNK group and MCD group were significantly differentiated, with *R*^2^*Y* = 0.998 and *Q*^*2*^ = 0.719, indicating that the OPLS-DA model was effective.

Variable importance in projection (VIP) (VIP ≥ 1) in OPLS-DA analysis and fold change (fold change ≥ 2 or ≤ 0.5) of univariate analysis were used to select differential lipid metabolites. As shown in Fig. 3d, MCD group showed 448 different lipid metabolites compared with MCS group, including 24 fatty acyls (FA), 297 glycerophospholipids (GP), 49 sphingolipids (SP), 15 sterol lipid (ST), 63 glycerides (GL). Compared with MCD group, HNK group showed 34 different lipid metabolites, 18 of them (including 17 FA and 1 GP) were down-regulated, and 16 of them (including 2 ST, 10 GP, 1 FA, 1 prenol lipids, 1 SP and 1 GL) were up-regulated (Fig. 3e).

In order to intuitively observe the change of the relative content of different lipid metabolites, we standardized the 34 screened significantly different lipid metabolites between MCD and HNK groups, and showed in the heat map (Fig. 3f). Compared with MCS group, 3 eicosanoids (12,13-EPOME, 9,10-DIHOME and 14(S)-HDHA) and 3 FFAs (16:2, 18:3 and 22:6) were significantly up-regulated, while TxB3, PA(18:0_22:6), SE(28:1_22:6) and 8 PEs (20:1_18:0, O-18:0_16:0, O-20:0_16:0, O-16:1_16:0, O-20:0_22:6, O-15:1_22:6, P-18:0_22:6 and P-16:0_16:0) were down-regulated in MCD group. HNK treatment significantly back-regulated the levels of these lipid metabolites. Furthermore, HNK treatment also increased the levels of taurolithocholic acid-3-sulfate (TLCA-3-S), PE(20:2_18:0), Coenzyme Q10, TG(O-20:0_16:0_18:1) and Cer(t18:0/24:0), and decreased the levels of PC(20:4_22:6) and 11 FFAs (14:0, 16:1, 17:1, 18:1, 19:1, 20:2, 20:3, 22:4, 22:5, 24:6 and 16:3), as compared to MCD mice.

KEGG pathway (www.kegg.jp/kegg/kegg1.html) enrichment analysis was used to determine the metabolic pathways related to the 34 different lipids mentioned above. Figure 3g showed that fatty acid biosynthesis and unsaturated fatty acid biosynthesis pathways were related to HNK alleviating lipid metabolism disorders in MCD mice.

### Effects of HNK on serum BAs levels and hepatic mRNA expression of BAs metabolism-related genes in NASH mice

The serum levels of 50 BAs were shown in Table S2 and the significant changes of serum BAs levels in mice were shown in Table 1. Compared with MCS group, the levels of 23-nor-deoxycholic acid (23-DCA), hyocholic acid (HCA), ursocholic acid (UCA), hyodeoxycholic acid (HDCA), deoxycholic acid (DCA), 7-ketodeoxycholic acid (7-KDCA), 3β-ursodeoxycholic acid (3β-UDCA) and 3-oxodeoxycholic acid (3-oxo-DCA) were increased in MCD group. HNK treatment markedly decreased the levels of 23-DCA, HDCA, glycocholic acid (GCA) and taurodeoxycholic acid (TDCA), as compared with MCD group.

**Table 1:** Levels of differential bile acids in serum of the tested mice.

BAs (ng/ml)	MCS	MCD	HNK
7-KDCA	717.72 ± 609.34	2875.19 ± 1749.38*	1924.01 ± 1137.45
DCA	223.43 ± 110.20	540.44 ± 301.67*	271.33 ± 96.50
TDCA	197.29 ± 229.70	308.34 ± 70.54	147.28 ± 29.11^##^
23-DCA	27.22 ± 17.66	277.30 ± 55.15**	97.73 ± 33.74^##^
HDCA	22.94 ± 10.95	44.70 ± 11.27**	24.53 ± 3.13^##^
UCA	19.27 ± 9.60	91.55 ± 58.73*	35.31 ± 23.46
3-oxo-DCA	13.85 ± 8.02	44.12 ± 29.71*	23.08 ± 17.75
HCA	13.50 ± 10.40	57.77 ± 41.79*	47.82 ± 26.85
GCA	6.43 ± 10.42	11.91 ± 1.42	6.18 ± 1.54^##^
3β-UDCA	3.30 ± 1.19	6.33 ± 2.97*	5.71 ± 2.72

(n = 7). **p < 0.01, *p < 0.05 vs MCS group; ##*p* < 0.01, #*p* < 0.05 vs MCD group. MCD, methionine- and choline-deficient diet; MCS, methionine- and choline-sufficient diet; HNK, honokiol.

**Figure 4:**
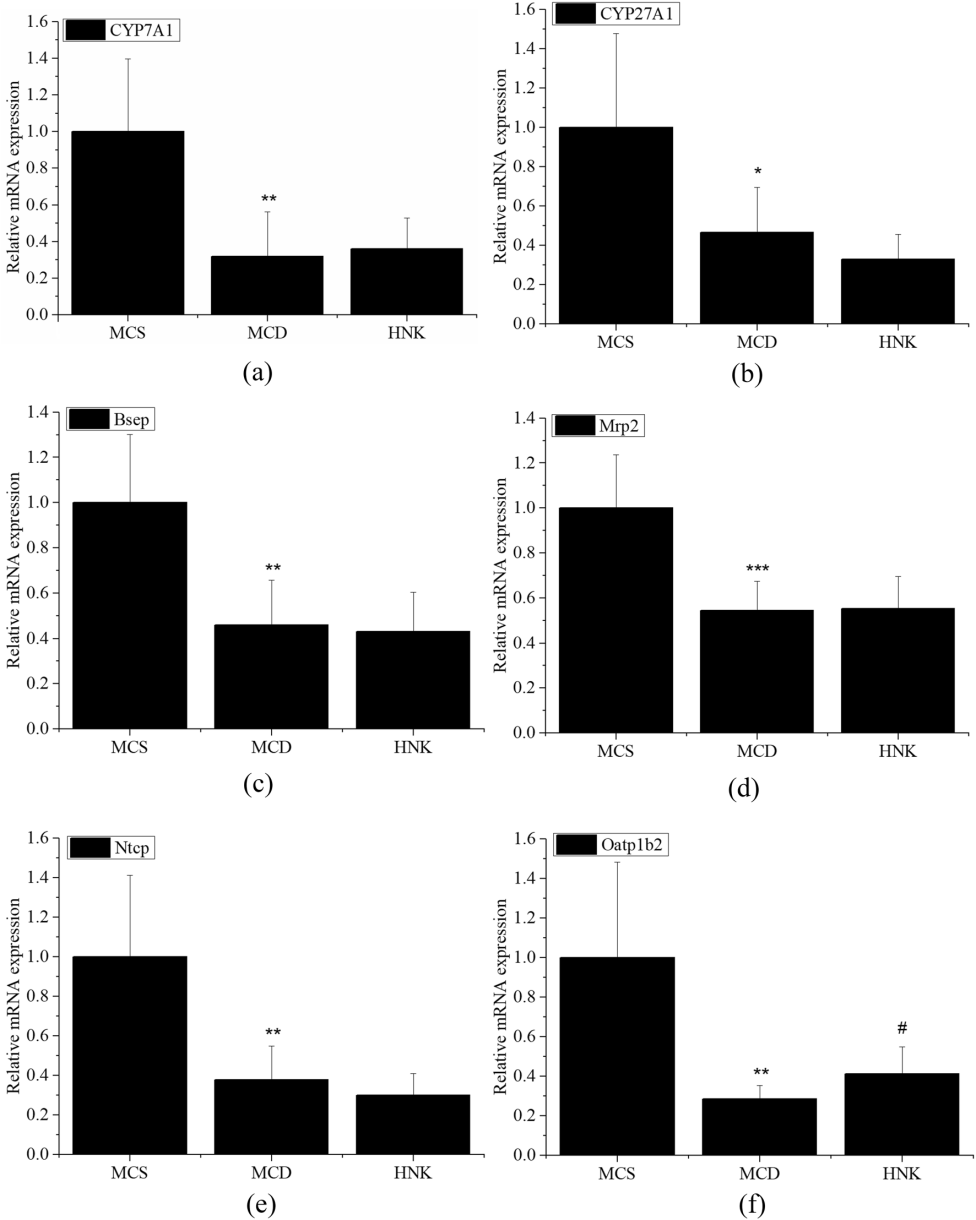
Effects of HNK on the mRNA expression of BAs metabolism-related genes in mice liver. The mRNA expression of *CYP7A1*(**a**), *CYP27A1*(**b**), *Bsep*(c), *Mrp2*(**d**), *Ntcp*(**e**) and *Oatp1b2*(**f**) were analyzed by RT-qPCR. The PCR products were normalized to *β-actin*. Data were expressed as mean ± SD (n = 7). ***p < 0.001, **p < 0.01, *p < 0.05 versus MCS group; #*p* < 0.05 versus MCD group. MCD, methionine- and choline-deficient diet; MCS, methionine- and choline-sufficient diet; HNK, honokiol.

The mRNA expression of BAs metabolism-related genes in mice liver were shown in Fig. 4. Compared with MCS group, the mRNA expression of *CYP7A1*, *CYP27A*1, *Bsep*, *Mrp2*, *Ntcp* and *Oatp1b2* in MCD group were significantly decreased. HNK treatment markedly increased *Oatp1b2* mRNA expression of MCD mice (Fig. 4f), whereas there were no significant effect on the mRNA expression of *CYP7A1*, *CYP27A1*, *Bsep*, *Mrp2* and *Ntcp* (Fig. 4a–e).

### Effects of HNK on the composition of GM in MCD mice

**Figure 5:**
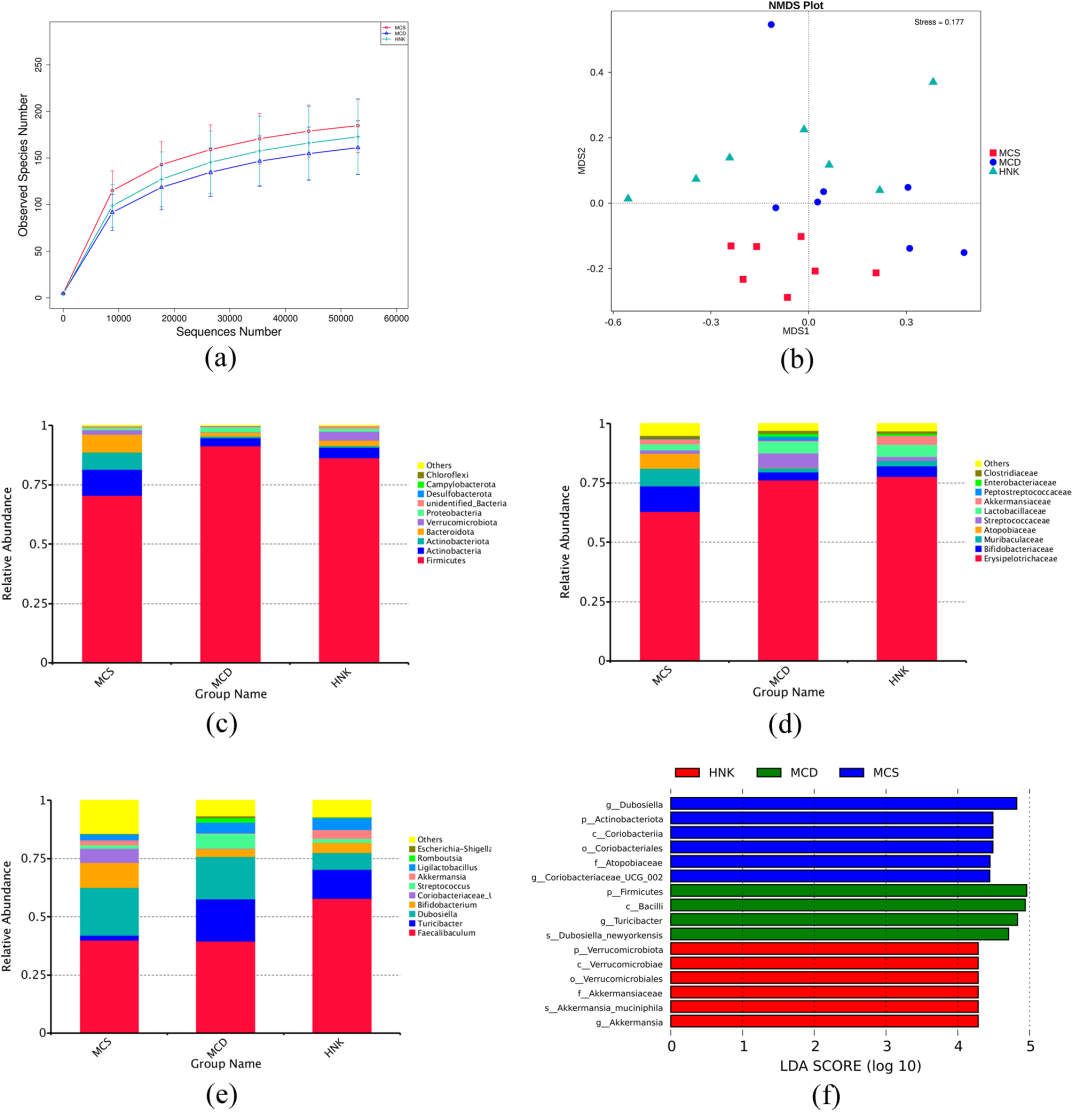
Effects of HNK on the composition of gut microbiota in the tested mice (n = 7). (**a**) Exponential dilution curve. (**b**) Non-metric multidimensional scaling (NMDS) on the OTU level. (**c**) Microbiota composition at the phylum level. (**d**) Microbiota composition at the family level. (**e**) Changes in gut microbiota of mice at the genus level. (**f**) LDA bar plot of LEFSe analysis on gut microbiota. MCD, methionine- and choline-deficient diet; MCS, methionine- and choline-sufficient diet; HNK, honokiol.

The Exponential dilution curve showed that the amount of 16Sr DNA sequencing data in this experiment was progressive and reasonable, and the sequencing depth was reliable (Fig. 5a). The statistical analysis of the Alpha diversity index of different samples under the 97% consistency threshold were shown in Table S3. There were no significant differences in Chao1, ACE, Simpson and Shannon indexes of GM among the three groups, indicating that HNK had no significant effect on the diversity of GM. Beta diversity analysis was applied to further study the composition of GM in each group, and non-metric multi-dimensional scaling (NMDS) showed significant differences in the composition of GM among the three groups (Fig. 5b).

The composition of GM of the tested mice at phylum, family and genus levels were shown in Fig. 5c–e. Compared with MCS group, the abundance of *Firmicutes* and *Turicibacter* were markedly increased, and the abundance of *Eggerthellaceae* was significantly decreased in MCD group. MCD mice treated by HNK significantly increased the relative abundance of *Ruminococcaceae*, *Caulobacteraceae* and *Brevundimonas*, and the abundance of *Firmicutes* and *Dubosiella* significantly decreased. Based on LEFSe analysis, species with LDA > 4 were considered as the set value, and the microbiota with statistical differences among three groups were screened out and showed in Fig. 5f.

### Spearman correlation analysis

**Figure 6:**
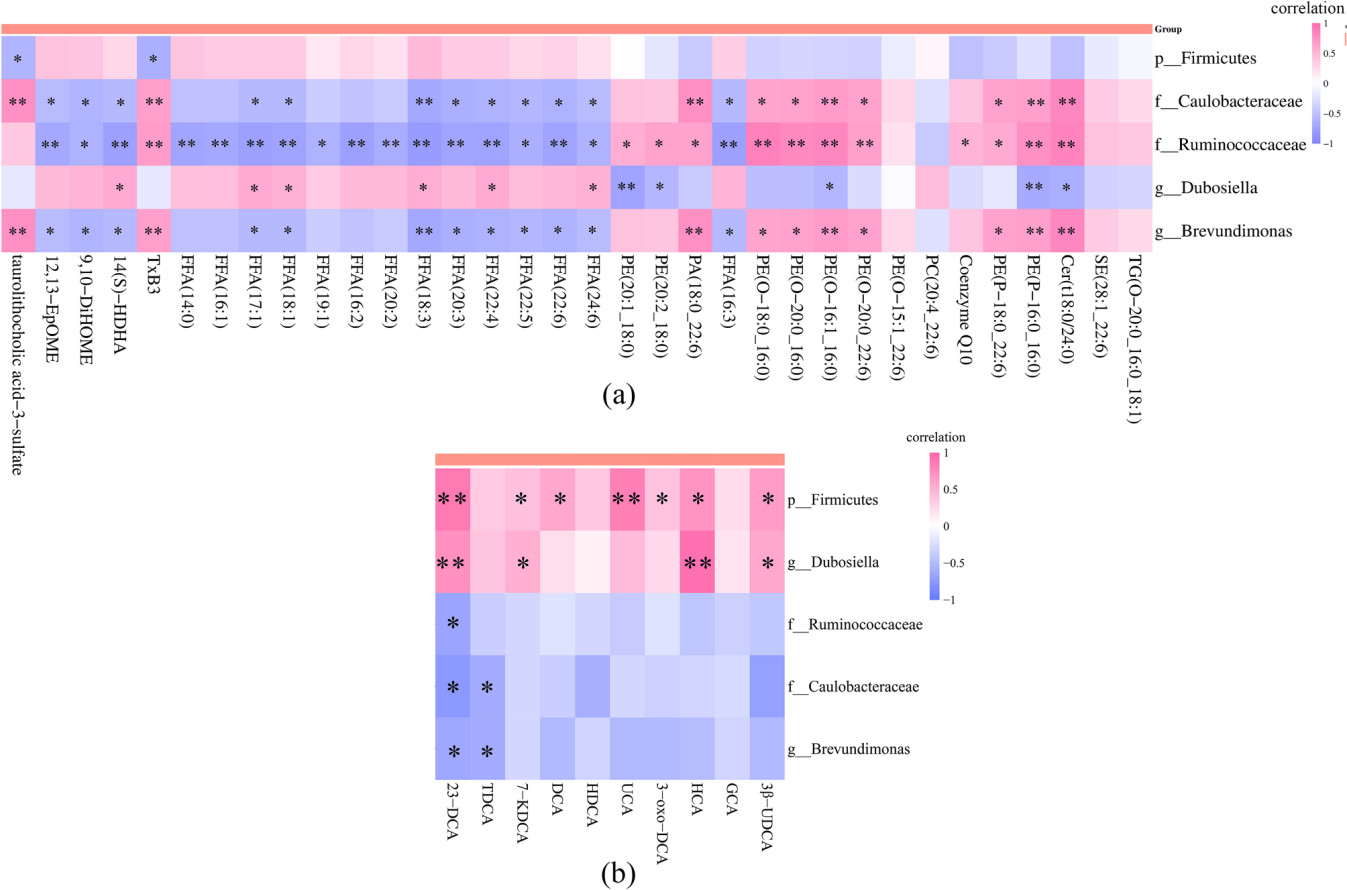
Spearman correlation analysis between gut microbiota and serum lipids or BAs in MCD mice. (**a**) Correlation between gut microbiota and serum lipids. (**b**) Correlation between gut microbiota and serum BAs. *Indicates that the *p* value of the correlation coefficient significance test is < 0.05, and **Indicates that the *p* value is < 0.01. MCD, methionine- and choline-deficient diet; MCS, methionine- and choline-sufficient diet; HNK, honokiol.

Spearman correlation analysis was used to explore the relationship between the abundance of GM and the contents of serum lipid or BAs significantly changed by HNK treatment in MCD mice. At phylum level, *Firmicutes* was negatively correlated with TLCA-3-S and TxB3 (Fig. 6a). At family level, *Ruminococcaceae* was negatively correlated with the levels of 14 FFAs (16:1, 16:2, 14:0, 18:1, 17:1, 22:4, 20:3, 18:3, 20:2, 19:1, 16:3, 22:6, 22:5, 24:6) (Fig. 6a), *Caulobacteraceae* was negatively correlated with 9 FFAs (18:1, 17:1, 22:4, 20:3, 18:3, 16:3, 22:6, 22:5 and 24:6) and positively related with 6 PEs (O-16:1_16:0, P-16:0_16:0, O-18:0_16:0, O-20:0_16:0, O-20:0_22:6 and P-18:0_22:6) (Fig. 6a). At genus level, *Dubosiella* was positively related with 4 FFAs (18:1, 17:1, 22:4, 18:3 and 24:6) and negatively correlated with multiple 4 PEs (20:1_18:0, 20:2_18:0, P-16:0_16:0 and O-16:1_16:0), *Brevundimonas* was negatively correlated with 2 oxidized lipids (12, 13-EPOME and 9,10-DiHOME) and 9 FFAs (18:1, 17:1, 16:3, 20:3, 22:4, 18:3, 22:5, 22:6 and 24:6) (Fig. 6a). At phylum level, *Firmicutes* was positively correlated with 23-DCA, 7-KDCA, DCA, UCA, 3-oxo-DCA, HCA, 3β-UDCA (Fig. 6b). At family level, *Caulobacteraceae* was negatively correlated with 23-DCA and TDCA, *Ruminococcaceae* was negatively correlated with 23-DCA (Fig. 6b). At genus level, *Brevundimonas* were negatively correlated with 23-DCA and TDCA, *Dubosiella* was positively related with 23-DCA, HCA, 7-KDCA, 3β-UDCA (Fig. 6b).

## Discussion

One of the sources of hepatic FAs is the input of FFAs in the blood. Excessive FFAs entering the liver will break the stable balance of hepatic TG and promote the occurrence of NAFLD^14^. If the body is continuously hit by high concentration of FFAs, it will not only interfere with multiple metabolic pathways and induce IR of multiple organs^15^, but also lead to fibrosis of liver tissues^16^. Phosphatidyl choline (PC) and phosphatidyl ethanolamine (PE) are the important lipid components of GP. Once the synthesis of PC in the body is blocked, the secretion and release of very low density lipoprotein cholesterol will be inhibited, so that TG in liver tissue cannot combine with it and transport out of liver, resulting in TG accumulation in liver tissue^17^. PE is the precursor of PC synthesis, and its level directly affects the degree of liver lipid accumulation. The levels of PC and PE in liver of NAFLD mice were down-regulated and further decreased with the progression of fatty liver disease^18^. Eicosanoids are the endogenous lipid signaling molecules produced by polyunsaturated fatty acids such as arachidonic acid under the catalysis of enzymes, which play important role in inflammation and tumor. 12,13-EPOME can increase the expression of pro-inflammatory cytokines in mouse macrophage RAW 264.7 and human colon cancer cell HCT-116^19^. 9, 10-DIHOME can not only destroy mitochondrial function by changing the integrity of cell intima and increasing the release of cytochromic C^20^, but also inhibit the respiratory burst of neutrophils, enhance cellular oxidative stress, dilate blood vessels, induce apoptosis and other reactions^21,22^. Additionally, thromboxane B3 (TXB3) is an eicosapentaenoic acid with anti-inflammatory effects^23^. The present work found that HNK reduced the levels of hepatic TG and MDA and alleviated liver inflammation of MCD mice. This process may be related to HNK-induced reduction of serum FFAs (14:0, 16:1, 17:1, 18:1, 19:1,16:2, 18:3, 22:6, etc.) and eicosanoids (12, 13-EPOME, 9, 10-DIHOME), as well as elevation of serum PEs (O-18:0:0, O-20:0:0, O-16:16:0, O-20:0:0:6, O-15:1:22:6, P-18:0:0:6, P-16:0:0), TXB3 and TLCA-3-S. In our study, KEGG pathway enrichment analysis showed that fatty acid and unsaturated fatty acid biosynthesis pathways were related to differential lipids induced by HNK. Previous studies have shown that HNK can alleviate lipid metabolism disorders of NASH by suppressing the expression of SREBP-1c, FAS, SCD-1 and promoting the phosphorylation of ACC, which are the key genes involved in fatty acid and unsaturated fatty acid biosynthesis pathways^11–13^. These results are consistent with the results of this study.

The abnormal changes of serum BAs are closely related to many chronic liver diseases. The levels of primary and secondary BAs in serum of NASH patients were significantly increased, and the severity of NASH was positively related with serum BAs levels^24^. The risk of NAFLD progressing to fibrosis is related to the ratio of secondary BAs to primary BAs and the concentration of conjugated BAs. The higher the ratio of secondary BAs to primary BAs and the higher the concentration of conjugated BAs, the higher the risk of fibrosis^25^. Hydrophobic BAs (such as DCA, 7-KDCA, 23-DCA and TDCA) are often regarded as toxic BAs due to their ability to activate death receptors and stimulate the production of pro-inflammatory mediators^26^, or due to their ability to weaken the enteric FXR/FGF19 signaling, thereby resulting in increased bile acid synthesis^27^. TDCA can damage mitochondria and induce apoptosis of HepG2 cells^28^. Additionally, the serum concentration of hydrophilic bile acid GCA in patients with NAFLD is significantly higher than that in healthy people^29^. In this study, HNK treatment reduced the levels of 10 BAs (HCA, UCA, DCA, 7-KDCA, 3β-UDCA, 3-oxo-DCA, 23-DCA, HDCA, TDCA and GCA) and ALT in serum of MCD mice, suggesting that HNK may reduce the risk of fibrosis and hepatotoxicity of MCD mice by regulating the metabolism of secondary BAs. The synthesis of primary BAs depends on the rate-limiting enzymes CYP7A1 and CYP27A1 in the liver. Bsep and Mrp2 are responsible for the transport of BAs out of the liver, NTCP and OATPs are responsible for uptake of BAs into the liver^30^. The present study showed that the mRNA expressions of CYP7A1 and CYP27A1 in MCD mice were significantly down-regulated, which is similar to human liver with NASH^31^. Notably, the mRNA expression of Bsep, Mrp2, Ntcp and Oatp1b2 in MCD mice liver were also significantly down-regulated, suggesting that the hepatotoxicity induced by MCD diet was probably due to the reduced BAs output from the liver, and the increased levels of serum BAs was mainly due to the decreased BAs input from the liver. After HNK treatment, only the mRNA expression of Oatp1b2 was up-regulated in MCD mice. Combined with the effect of HNK on serum BAs of MCD mice, we speculated that the improvement of HNK on BA metabolism disorder in MCD mice resulted from, on one hand, its effect on liver Oatp1b2 expression, and on other hand, its effect on the composition and abundance of GM.

The GM in NAFLD patients, obese people, ob/ob and db/db mice is characterized by a higher proportion of *Firmicutes* and *Bacteroidetes*, an increased *Proteobacteria* producing endotoxins, and a decreased immunohomeostasis-related bacteria^32,33^. *Firmicutes* is harmful bacteria, and its main metabolites acetic acid and propionic acid are substrates for liver gluconeogenesis and adipose formation^34^. *Ruminococcaceae* is a probiotics that can degrade a variety of polysaccharides and fibers to produce SCFAs such as butyrate, its abundance is significantly negatively correlated with alcoholic cirrhosis, non-alcoholic liver fibrosis, and increased intestinal permeability^35–37^. Effective regulation of GM has a positive impact on decreasing liver fat accumulation and alleviating NAFLD^38,39^. Our results showed that HNK markedly decreased the abundance of *Firmicutes* and *Dubosiella*, and increased the abundance of *Ruminococcaceae*, *Caulobacteraceae* and *Brevundimonas*, suggesting that HNK may improve MCD mice by regulating the abundance of GM mentioned above. Notably, the oral bioavailability of HNK in rats was reported to be only 5.3% due to extensive first-pass metabolism and low absorption^40^, suggesting that the regulatory effect of HNK on lipids and bile acids may be realized through the regulation of gut microbiota.

In the occurrence and development of NASH, lipids, BAs and GM are closely related. Secondary BAs are mainly metabolized and generated by BA hydrolase encoded by intestinal microorganisms. Changes in intestinal microbial community will change the expression level of BA hydrolase, thus affecting the composition of the host BA pool, and thereby affecting the signal transmission of BAs^41^. GM regulates the synthesis and metabolism of liver BAs through FXR negative feedback, and the composition of BA pools also affects the composition of GM^42^. GM is also closely related to lipid metabolism. Obese people have a higher ratio of *Firmicutes*/*Bacteroidetes* in the intestinal tract, which has a higher ability to obtain energy from food^43^. Our results showed that the abundance of *Ruminococcaceae* was negatively correlated with the serum levels of 14 FFAs and 23-DCA, *Firmicutes* was negatively correlated with TLCA-3-S and TxB3, and positively related with 23-DCA, 7-KDCA, DCA, UCA, 3-oxo-DCA, HCA, 3β-UDCA. The correlation analysis results showed that the up-regulation effect of HNK on the beneficial bacteria *Ruminococcaceae* of MCD mice was well coordinated with its regulating effect on the levels of serum beneficial lipids, harmful lipids and bile acids. This coordination of regulation is also shown in its effects on the abundance of intestinal harmful bacteria *Firmicutes* and serum lipid and bile acid levels.

Our results suggested that HNK ameliorated the disorder of lipid and bile acid metabolism, up-regulated the abundance of intestinal probiotics and down-regulated the abundance of harmful bacteria in MCD mice. These may be closely related to its improvement effect of NASH. BAs metabolism runs through the enterohepatic circulation, and many regulatory factors in the gut, such as FXR and a variety of transporters, are directly involved. In this paper, only the effects of HNK on BAs homeostasis related enzymes and transporters in MCD mice were studied, and there was a lack of intestinal research data. In subsequent study, the effect of HNK on BAs metabolism related genes in the gut of mice should be further explored, and it should be linked with the effect of HNK on the alteration of GM, to determine whether the regulatory effect of HNK on BAs is related to the alteration of the abundance and composition of GM, and to provide a more accurate reference for the preclinical study of HNK against NASH.

## Materials and methods

### Main drugs and reagents

HNK (CAS NO. 35354–74-6, HPLC ≥ 98%) and obeticholic acid (OCA) (CAS NO. 459789–99-2, HPLC ≥ 98%) were from Shanghai Yuanye Biotechnology Co.Ltd. Methionine-choline deficient (MCD) and methionine-choline sufficient (MCS) diet were from Nantong Trophy Feed Technology Co. Ltd. (Jiangsu, China). Alanine aminotransferase (ALT), aspartate aminotransferase (AST), triglyceride (TG), malondialdehyde (MDA) detection kits were obtained from Nanjing Jiancheng Institute of Biological Engineering (Nanjing, China). The standards of 12:0 Lyso PC, Cer (D18:1/4:0), PC (13:0/13:0), DG (12:0/12:0) and TG (17:0/17:0/17:0) were purchased from Avanti/ ZzStandard. The standards of 50 bile acids were purchased from CNW/IsoReag. Phusion® High Fidelity PCR Master Mix with GC Buffer was purchased from New England Biolabs. ReverTra Ace qPCR RT Master Mix with gDNA Remover kit was from Toyobo Co., LTD. (Life Science Department Osaka, Japan). SYBR Green I fluorescent quantitative PCR kit was from Bio-Rad (Hercules, CA, USA).

### Animal experiment

SPF male C57BL/6 mice weighing 19–23 g and aged 6–8 weeks were obtained from China Three Gorges University (Hubei, China). All mice were kept in the SPF room of Hubei University. Water and food were provided ad libitum during the feeding process. Subsequent experiments were performed after 7 days of adaptive feeding. Mice were randomly divided into 4 groups, with 7 mice in each group. Normal control group was fed by MCS diet (methionine-choline free amino acid premix, 17.57%; methionine, 0.80%; choline chloride, 0.20%; sucrose, 44.17%; corn starch, 15%; dextrin, 5%; cellulose, 3%; corn oil, 10%; mineral mix, 5.25%)and administered simultaneously with 0.5% CMC-Na solution by gavage. Model group, HNK group and OCA group were fed MCD diet (methionine-choline free amino acid premix, 17.57%; sucrose, 44.17%; corn starch, 15%; dextrin, 5%; cellulose, 3%; corn oil, 10%; mineral mix, 5.25%) and administered respectively simultaneously with 0.5% CMC-Na solution, HNK (20 mg/kg/d) or OCA (6.5 mg/kg/d) by gavage once a day for 8 weeks. Weight was measured once a week. After the last administration, mice were fasted for 12 h before being sacrificed by neck removal.

All animal experiments were carried out in accordance with the recommendations in the Guide for the Care and Use of Laboratory Animals of the National Institutes of Health and followed the recommendations in the ARRIVE guidelines. The protocol was approved by the Experimental Animal Ethics Committee of Hubei University (Protocol Number: 20220039). All efforts were made to minimize suffering.

### Histological examination and biochemical analysis

Fresh mouse livers of about 1 cm^3^ in size were dissected and immediately fixed in 4% paraformaldehyde for 24 h. Fixed tissues were embedded in paraffin and 4-µm sections were prepared and respectively stained with hematoxylin–eosin (HE), masson trichromatic staining or oil red O staining for morphological observations.

The levels of ALT and AST in serum, as well as TG, MDA in liver homogenate (liver-saline, 1:9, *w/v*) were determined by using commercial test kits according to the manufacturer's instructions.

### Sample preparation

50 μL serum of each mouse was mixed with 1 mL internal standard lipid extract (Methyl tert-butyl ether: Methanol = 3:1, V/V), vortex for 15 min, then add 200μL pure water, vortex for 1 min, centrifuge at 4 °C (12,000 rpm/min) for 10 min. Add 200μL acetonitrile/isopropyl alcohol (10/90, V/V) to the 200μL supernatant concentrate, vortex for 3 min, centrifugation at 12,000 r/min for 3 min. The supernatant was used for sample analysis. Identification of lipids and bile acids was based on self-built database MWDB (Metware database).The quality control sample (QC) is prepared by mixing the extracts of the samples in equal quantities. During the analysis, one QC was inserted for every 10 samples. The stability of the instrument during the project inspection can be judged by the overlapping display analysis of the total ion flow chromatogram (TIC) of the same quality control sample.

### Serum lipomic analysis

Serum lipids of mice were detected by ExionLC AD UPLC-QTRAP (SCIEX, USA).The chromatographic column was Thermo Accucore™ C30(2.1 × 100 mm, 2.6 um). Phases A consist of acetonitrile/water (60/40, v/v) (containing 0.1% formic acid and 10 mmol/L ammonium formate). Phases B consist of acetonitrile/isopropanol (10/90, v/v) (containing 0.1% formic acid and 10 mmol/L ammonium formate). Gradient elution procedure was as follows: 0–2 min, 30%B; 2–4 min, 60%B; 4–9 min, 85%B; 9–14 min, 90%B; 14–15.5 min, 95%B; 17.3–17.5 min, 20%B; 17.5–20 min, 20%B. The flow rate was 0.35 mL/min, the column temperature was 45 °C, and the injection volume was 2 μL. The positive and negative ion scanning modes of electrospray ionization were adopted for mass spectrometry detection. The ion source temperature was 500 °C, and the mass spectrometry voltage in positive/negative ion mode was 5500 V/− 4500 V. The ion source GAS1 (GS1), ion source GAS2 (GS2), curtain Gas (CUR) were 45, 55 and 35 psi, respectively.

The LC–MS data was preprocessed by Analyst 1.6.3 software, and the processed data was analyzed by R software (www.r-project.org/) for multivariate statistical analysis, including Principal Component Analysis (PCA) and Orthogonal Partial Least Squares Discriminant Analysis (OPLS-DA). According to Variable Importance (VIP) value and fold change value, abnormal lipid components were screened out. The abnormal lipid components were imported into KEGG (Kyoto Encyclopedia of Genes and Genomes) database for metabolic pathway enrichment analysis.

### Serum BAs analysis

Serum BAs of mice were detected by ExionLC AD UPLC-QTRAP 6500 + (SCIEX, USA). The chromatographic column was Waters ACQUITY UPLC HSS T3 C18 (1.8 µm, 100 mm × 2.1 mm). Mixed mobile phase was consisted of A (ultra-pure water, containing 0.01% acetic acid and 5 mmol/L ammonium acetate) and B (acetonitrile, containing 0.01% acetic acid). The preset multi-response monitoring mode at negative ion of ESI were adopted for mass spectrometry detection. The ion source temperature, mass spectrometry voltage and curtain gas were 550 °C, − 4500 V and 35 psi, respectively.

### 16S rDNA analysis of GM

The ileal content from each mice was collected to extract the total bacterial genomic DNA by CTAB method. The V4 hypervariable region of the bacterial 16S rDNA gene was amplified using specific primers with Barcode (515F and 806R), Phusion® High-fidelity PCR Master Mix with GC Buffer (New England Biolabs) and high-fidelity enzyme. TruSeq® DNA PCR-free sample preparation kit was used for library construction. The constructed library was quantified by Qubit and Q-PCR, then sequencing was performed using NovaSeq6000. Pair-end reads was spliced after sequencing according to overlap relation and barcode sequence was removed. Effective sequence was generated after statistical analysis of number and length distribution of sequences, and the percentage of effective sequence was optimized. Sequences with more than 97% similarity were grouped into the operational taxonomic units (OTUs) for subsequent analysis.

### RT-Qpcr

Trizol reagent was used to extract the total RNA from liver tissues (50–100 mg) of each mouse. 1.5 μg total RNA was reverse transcribed into cDNA using ReverTra Ace® qPCR RT kit (Toyobo, Japan). To analyze the relative mRNA expression of the genes, we use SYBR Green fluorescent quantitative PCR kit and CFX Connect™ Real-Time System (BIO-RAD,USA). Relative mRNA expression levels were calculated using the comparative Ct method (2^−ΔΔCt^) normalized to β-actin. Table S1 showed the primer sequences of each tested gene.

### Statistical analysis

Data were analyzed by SPSS 25.0 and expressed as mean ± SD (standard deviation). Statistical analyses were performed using one-way analysis of variance (ANOVA), followed by student’s t-test. Values were considered significant at *P* < 0.05.

### Ethical approval

This study was carried out in accordance with the recommendations in the Guide for the Care and Use of Laboratory Animals of the National Institutes of Health and followed the recommendations in the ARRIVE guidelines. The protocol was approved by the Experimental Animal Ethics Committee of Hubei University (Protocol Number: 20220039).

### Supplementary Information

Supplementary Information.

## Data Availability

The data that supports the findings of this study are available in the article and the supplementary material of this article.

## Abbreviations

HNK: Honokiol
NASH: Non-alcoholic steatohepatitis
MCD: Methionine–choline deficiency
MCS: Methionine–choline sufficient
BA: Bile acid
GM: Gut microbiota
HE: Hematoxylin–eosin
OCA: Obeticholic acid
ALT: Alanine aminotransferase
AST: Aspartate aminotransferase
TG: Triglyceride
MDA: Malondialdehyde
PCA: Principal component analysis
OPLS-DA: Orthogonal partial least squares discriminant analysis
CYP7A1: Cholesterol 7-alpha-hydroxylase
CYP27A1: Sterol 27-hydroxylase
Bsep: Bile salt export pump
Mrp2: Multidrug resistance-associated protein 2
Ntcp: Na^+^ taurocholate cotransporting polypeptide
Oatp1b2: Organic anion transporting polypeptide 1b2
