# Supplementary material for: Honokiol affects the composition of gut microbiota and the metabolism of lipid and bile acid in methionine-choline deficiency diet-induced NASH mice
Source: Sci Rep. 2023 Sep 14;13:15203. doi: 10.1038/s41598-023-42358-w (PMC10502053; doi:10.1038/s41598-023-42358-w)
Supplement: Supplementary file 1 — Supplementary Information. [file 41598_2023_42358_MOESM1_ESM.pdf]

Honokiol affects the composition of gut microbiota and the metabolism of lipid and bile acid in methionine-choline deficiency diet-induced NASH mice

Ting Zhai, Junjun Wang, Yong Chen*

Hubei Province Key Laboratory of Biotechnology of Chinese Traditional Medicine, National Local Joint Engineering Research Center of High-throughput Drug Screening Technology, State Key Laboratory of Biocatalysis and Enzyme Engineering, Hubei University, Wuhan 430062, China.

***Corresponding author:**
Yong Chen
cy101610@qq.com

Table S1: Primer sequences used for RT-qPCR

Name	Accession number		Sequences
β-actin	NM_007393	Forward	5´-AGAGGGAAATCGTGCGTGAC-3´
		Reverse	5´-CAATAGTGATGACCTGGCCGT-3´
CYP7A1	NM_007824	Forward	5´-GGGAATGCCATTTACTTGGA-3´
		Reverse	5´-GTCCGGATATTCAAGGATGC-3´
CYP27A1	NM_024264	Forward	5´-CTATGTGCTGCACTTGCCC-3´
		Reverse	5´-GGGCACTAGCCAGATTCACA-3´
Bsep	NM_021022	Forward	5´-CCAGAACATGACAAACGGAA-3´
		Reverse	5´-AAGGACAGCCACACCAACTC-3´
Mrp2	NM_013806	Forward	5´-TCCAGGACCAAGAGATTTGC-3´
		Reverse	5´-TCTGTGAGTGCAAGAGACAGGT-3´
Ntcp	NM_011387	Forward	5´-AGGGGGACATGAACCTCAG-3´
		Reverse	5´-TCCGTCGTAGATTCCTTTGC-3´
Oatp1b2	NM_020495	Forward	5´-ACCAAACTCAGCATCCAAGC-3´
		Reverse	5´-TAGCTGAATGAGAGGGCTGC-3´

Table S2 Levels of bile acids in serum of the tested mice

BAs（ng/ml）	MCS	MCD	HNK
7-KDCA	717.72±609.34	2875.19±1749.38*	1924.01±1137.45
DCA	223.43±110.20	540.44±301.67*	271.33±96.50
TDCA	197.29±229.70	308.34±70.54	147.28±29.11$^{\#\#}$
23-DCA	27.22±17.66	277.30±55.15**	97.73±33.74$^{\#\#}$
HDCA	22.94±10.95	44.70±11.27**	24.53±3.13$^{\#\#}$
UCA	19.27±9.60	91.55±58.73*	35.31±23.46

3-oxo-DCA	13.85±8.02	44.12±29.71*	23.08±17.75
HCA	13.50±10.40	57.77±41.79*	47.82±26.85
GCA	6.43±10.42	11.91±1.42	6.18±1.54##
3β-UDCA	3.30±1.19	6.33±2.97*	5.71±2.72
DHCA	1.35±0.55	2.46±0.66**	2.07±0.33
CDCA	174.90±115.66	492.11±368.43	562.00±156.60
ω-MCA	1718.36±1049.35	4055.40±2399.02	2516.06±1136.71
β-MCA	665.49±874.10	2287.85±1722.11	1551.97±721.77
α-MCA	82.50±113.35	205.27±123.40	117.16±52.36
UDCA	58.77±22.91	139.24±102.53	76.76±53.00
MDCA	11.80±11.65	16.70±13.75	7.97±3.64
LCA	16.49±4.40	25.18±3.01**	26.86±5.52
CA	997.81±1088.62	1947.89±789.86	1689±718.98
3β-DCA	5.02±1.54	26.40±22.64	8.62±5.13
3-oxo-CA	3.18±3.86	5.43±3.46	3.89±2.18
GCDCA	0.25±0.15	0.35±0.05	0.36±0.12
GLCA	1.80±0.16	1.93±0.21	1.77±0.62
Tβ-MCA	6483.13±11251.70	8271.16±1923.95	9959.98±4334.27
TUDCA	183.87±266.98	256.93±51.08	246.91±76.15
TLCA	10.87±8.55	16.35±2.71	12.38±4.92
TCDCA	386.30±637.60	429.11±104.93	590.60±175.49
TCA	6918.00±12057.00	5687.88±862.85	5795.15±1901.50

12-KLCA	___	___	___
NCA	___	___	___
DLCA	___	___	___
3β-CA	___	___	___
6,7-DKLCA	___	___	___
12-oxo-CDCA	___	___	___
3β-HDCA	___	___	___
IALCA	___	___	___
IDCA	___	___	___
ILCA	___	___	___
LCA-3S	___	___	___
7-KLCA	___	___	___
7,12-DKLCA	___	___	___
CDCA-3Gln	___	___	___
GLCA-3S	___	___	___
GHCA	___	___	___
GDHCA	___	___	___
GDCA	___	___	___
GUDCA	___	___	___
THCA	___	___	___
TDHCA	___	___	___
TLCA-3S	___	___	___

n=7. **p < 0.01, *p < 0.05 vs MCS group; ##p < 0.01, #p < 0.05 vs MCD group. MCD, methionine- and choline-deficient diet; MCS, methionine- and choline-sufficient diet; HNK, honokiol. __: not quantified.

Table S3 Effects of HNK on the Alpha diversity of GM in the tested mice

	Chao1	ACE	Shannon	Simpson
MCS	201.01±11.93	205.26±13.14	2.95±0.24	0.74±0.04
MCD	183.66±10.90	187.49±13.73	2.65±0.13	0.69±0.03
HNK	189.29±32.37	197.66±16.69	2.31±0.15	0.60±0.04

Data were expressed as mean±SD (n=7). MCD, methionine- and choline-deficient diet; MCS, methionine- and choline-sufficient diet; HNK, honokiol.
